# Hesperetin Induces Autophagy and Delayed Apoptosis by Modulating the AMPK/Akt/mTOR Pathway in Human Leukemia Cells In Vitro

**DOI:** 10.3390/cimb45020102

**Published:** 2023-02-13

**Authors:** Ching-Yeh Lin, Ya-Hui Chen, Ying-Chih Huang

**Affiliations:** 1 Department of Internal Medicine, Changhua Christian Hospital, Changhua 50006, Taiwan; 2 Women’s Health Research Laboratory, Changhua Christian Hospital, Changhua 50006, Taiwan; 3 Hematology & Oncology Research Laboratory, Changhua Christian Hospital, Changhua 50006, Taiwan

**Keywords:** leukemia, hesperetin, apoptosis, autophagy, AMPK/Akt/mTOR

## Abstract

Background: Hesperetin has been reported to have anticancer properties. However, the molecular mechanisms underlying its action on leukemia cells remain unclear. This in vitro study evaluated the possible mechanisms of hesperetin in leukemia cells (HL-60 and U937). Methods: Cell viability was evaluated using a cell counting kit-8 (CCK-8) assay. Apoptosis and autophagy assays were conducted through annexin V/PI staining and acidic vesicular organelle (AVO) staining. Cell cycle analysis was conducted through propidium iodide (PI) and flow cytometry. The expression of proteins related to apoptosis and autophagy, including cleaved-PARP-1, Bcl-2, Bax, LC3-I/II, Beclin-1, Atg5, p62, phospho-AMPK, AMPK, phospho-mTOR, mTOR, phospho-Akt, and Akt, in human leukemia cells were evaluated using Western blotting. Results: Hesperetin dose-dependently inhibited leukemia cell viability. However, we found a low degree of apoptosis and cell cycle arrest induced by hesperetin in U937 cells. These findings imply the presence of additional mechanisms modulating hesperetin-induced cell death. Next, we evaluated autophagy, the possible mechanism modulating cell death or survival, to clarify the underlying mechanism of hesperetin-induced cell death. Hesperetin also dose-dependently increased the ratio of LC3II/I, Atg5, and Beclin 1 and decreased p62. Moreover, 3-methyladenine (3-MA) and bafilomycin A1 (Baf-A1) inhibited hesperetin-induced autophagy. We suggest that hesperetin can protect cancer cells during the transient period and may extend survival. Furthermore, a decrease in p-mTOR and p-Akt expression and an increase in p-AMPK expression were observed. Collectively, these findings suggest that hesperetin induces autophagy by modulating the AMPK/Akt/mTOR pathway. Conclusion: Hesperetin promoted cell death in the human leukemic cell line U937 by inducing a low degree of slight apoptosis, cell cycle arrest, and autophagy. It is therefore a potential adjuvant to antileukemia therapy and may be combined with other chemotherapeutic drugs to reduce chemoresistance and side effects.

## 1. Introduction

Leukemia is a major public health problem worldwide and is also the leading cause of death in Taiwan [1,2]. The main treatment for acute myeloid leukemia in adults is chemotherapy. However, the use of more intensive chemotherapy regimens has increased the treatment response but has also increased the incidence of side effects, such as low white blood cell counts. Therefore, effective adjuvant treatment is urgently required to reduce possible side effects.

Hesperetin (3′, 5′, 7′-trihydroxy-4-methoxyflavone) is a natural compound derived from citrus fruits that has anticancer, antioxidant, anti-inflammatory, antiplatelet activities, and antiviral properties [3,4,5,6]. Its antitumor effect has been reported in various cancers, such as breast cancer, cervical cancer, and colon cancer; this effect occurs through the inhibition of cancer cell proliferation by activating apoptosis and inhibiting angiogenesis [7,8,9]. Most interestingly, it gained attention for its anti-COVID-19 activities [10]. For example, hesperetin increased the levels of p53 and cyclin-dependent kinase inhibitors and decreased the levels of certain cyclins and cyclin-dependent kinases, which have been linked to cell cycle arrest and cytostatic effects [11]. In addition, hesperetin may maintain the autophagic balance, resulting in various benefits, such as neuroprotective, anticancer, antidiabetic properties, and antiviral properties. As a natural compound, hesperetin offer good feasibility in clinical application in the future.

However, the study in the anticancer effect of hesperetin on acute myeloid leukemia is rare. Here, the aim of this study was to investigate the molecular mechanisms underlying hesperetin-induced cytotoxicity in leukemia cells. We demonstrated that hesperetin treatment can lead to a low degree of apoptosis mediated through autophagy in U937 cells. Hesperetin also induced autophagy through the AMPK/mTOR pathway, leading to cell death.

## 2. Materials and Methods

### 2.1. Chemicals and Antibodies

Hesperetin (Cat. No. 520-33-2) was purchased from ChemFaces Company (ChemFaces, Wuhan, China) and a 100 mM stock solution was prepared in DMSO (0.1% *v*/*v* final concentration) and stored at −20 °C. 3-Methyladenine (3-MA, an autophagy inhibitor) was purchased from Sigma Chemical Co. (St. Louis, MO, USA), and bafilomycin A1 (Baf-A1) was purchased from Santa Cruz Biotechnology (Santa Cruz, CA, USA). Polyclonal anti-p-AMPKα (Thr172; #2535), polyclonal anti-AMPKα (D63G4) (#5832), polyclonal anti-p-mTOR (Ser2448; #5536), polyclonal anti-mTOR (7C10) (#2983), polyclonal anti-cleaved PARP (#9532), polyclonal anti-Beclin-1 (#3495), polyclonal anti-SQSTM1/p62 (#5114), polyclonal anti-p-Akt (Ser4723; #4060), polyclonal anti-p-Akt (#9272), polyclonal anti-Atg5 (#2630), monoclonal anti-Bcl-2 (#15071), monoclonal anti-Bax (#5023), and monoclonal anti-GAPDH (#0411) antibodies were purchased from Cell Signaling Technology (Danvers, MA, USA). We also purchased polyclonal-anti-LC-3B (#L7543; Sigma, UK), goat anti-rabbit IgG secondary antibodies (#NA934; GE Healthcare Life Sciences, Chalfont, UK), and IgG HRP-conjugated secondary antibody (polyclonal anti-mouse; #115-035; 1:5000; Jackson ImmunoResearch Laboratories, UK).

### 2.2. Cell Lines and Treatments

The human leukemic monocyte lymphoma cell line U937 and the promyelocytic leukemia cell line HL-60 were obtained from the Bioresource Collection and Research Center (Hsinchu, Taiwan; derived from ATCC CRL-1593.2 and ATCC CCL-240). The cells were cultured in RPMI-1640 and Iscove’s modified Dulbecco’s medium containing 10–20% fetal bovine serum supplemented with 100 U/mL penicillin/streptomycin (P/S) at 37 °C in a humidified atmosphere of 5% CO_2_.

### 2.3. Cell Viability Assay

The U937 and HL-60 cell lines were seeded in 96-well plates at a density of 4 × 10^4^ cell/well and were incubated with various concentrations of hesperetin for 24 and 48 h. Subsequently, 10 µL of CCK-8 solution was added to each well at 37 °C, and optical density (OD) was measured at 450 nm on a microplate reader (Bio-Rad 680). The results are presented as a percentage of the values measured for the untreated control cells. The relative cell viability of the controls was calculated as

(OD_test_/OD_control_) × 100%

The results were shown as IC_50_ values, i.e., the concentration that inhibited 50% of cell growth, which was enumerated by graphical extrapolation using GraphPad prism software (version 9.0, GraphPad Software Inc., San Diego, CA, USA). Each experiment was performed at least three times and in duplicate.

### 2.4. Analysis of Apoptosis

An annexin V-based kit (Apoptest-FITC Kit, Dako, Glostrup, Denmark) was used to estimate the extent of apoptosis in U937 cells after treatment with hesperetin (0, 12.5, 25, 50, and 100 µM) for 24 h. U937 cells at a density of 5 × 10^5^ cells/well were collected and washed with cold PBS. The cell suspensions were stained with annexin V-FITC and propidium iodide (PI) for 10 min at room temperature in the dark. Fluorescence-activated cell sorting cater-plus flow cytometry (Becton Dickinson, Mountain View, CA, USA) was used to analyze early apoptotic cells and late apoptotic or necrotic cells; at least 10,000 cells per group were counted.

### 2.5. Cell Cycle Assay

U937 cells (5 × 10^5^) were treated with hesperetin (0, 12.5, 25, 50, and 100 µM) for 24 and 48 h. Next, the cells were collected and washed twice with cold PBS and fixed with 75% ice-cold ethanol at −20 °C overnight. After fixation, the cells were washed twice with cold PBS and centrifuged at 1200 rpm for 5 min at 4 °C. The cells were stained with 500 µL of PI/RNase staining buffer (#550825, BD Pharmingen; BD Biosciences, Franklin Lakes, NJ, USA), followed by incubation in the dark at room temperature for 30 min. Finally, the cells were analyzed using the FACSCalibur system.

### 2.6. Analysis of Autophagy

Acidic intracellular compartments were evaluated using acidic vesicular organelle (AVO) staining. U937 cell staining was performed as previously described [11,12,13]. To examine the degree of autophagy in U937 cells, the cells were treated with hesperetin (0, 12.5, 25, 50, and 100 µM) for 24 h. The cell pellets were washed twice with cold PBS and centrifuged at 1200 rpm for 5 min at 4 °C. Acridine orange (Polysciences, Warrington, PA, USA) was added at a final concentration of 1 mg/mL for 15 min at 37 °C in the dark. The FACSCalibur system (Becton Dickinson) was used to analyze the autophagic cells. Cell viability assay was used to evaluate the inhibition of cell proliferation potentials of 3-Methyladenine (3-MA) and bafilomycin A1 (Baf-A1). Also, U937 cells were seeded in 96-well plates (4 × 10^4^ cells/well) and incubated at 37 °C for 24 h, pretreated with or without 3-MA (2 mmol/L) for 2 h, and then stimulated with various concentrations of hesperetin (0, 25, 50, and 100 µM). The wells without 3-MA or Baf-A1 and hesperetin addition were used as controls. Each concentration was replicated three times. Following incubation for 24 h, 10 µL of CCK-8 (Sigma-Aldrich) was added to each well, and the cells were reincubated at 37 °C for 2 h. Absorbance was measured using a multifunctional microplate reader (Molecular Devices, Sunnyvale, CA, USA) at 450 nm.

### 2.7. Western Blot Assay

The U937 cell line was lysed in 1× RIPA buffer (N653, Amresco, Solon, OH, USA) with 10% proteasome inhibitor. All cell extracts were cleared at 13,000 rpm for 30 min in a microcentrifuge at 4 °C. Proteins were separated using 10% SDS-PAGE and were transferred to a PVDF membrane. The membrane was blocked in 5% nonfat dry milk in PBS buffer with Tween 20. Immunostaining was performed using the following antibodies: monoclonal anti-p-AMPKα (Thr172; #2535), monoclonal anti-AMPKα (#5832), polyclonal anti-p-mTOR (Ser2448; #5536), polyclonal anti-mTOR (7C10) (#2983), polyclonal anti-cleaved PARP (#9532), polyclonal anti-Beclin-1 (#3495), polyclonal anti-SQSTM1/p62 (#5114), polyclonal anti-p-Akt (Ser4723; #4060), polyclonal anti-p-Akt (#9272), polyclonal anti-Atg5 (#2630), monoclonal anti-Bcl-2 (#15071), monoclonal anti-Bax (#5023), and monoclonal anti-GAPDH (#0411) obtained from Cell Signaling Technology; and polyclonal-anti-LC-3B (#L7543; Sigma). The secondary antibodies were goat anti-rabbit IgG (#NA934, GE Healthcare Life Sciences) and IgG HRP-conjugated secondary antibodies (polyclonal anti-mouse; #115-035; 1:5000; Jackson ImmunoResearch Laboratories, London, UK). An ECL Western blotting reagent (GE Healthcare Life Sciences, Massachusetts, MA, USA) was used for protein detection.

### 2.8. Statistical Analysis

The data were analyzed using GraphPad Prism v9.0 (San Diego, CA, USA), and *p* < 0.05 was considered statistically significant. Data between the two groups were compared using an unpaired two-tailed Student’s *t* test. All values are expressed as mean ± SD.

## 3. Results

### 3.1. Hesperetin Reduces Human Leukemic Cell Viability

**Figure 1:**
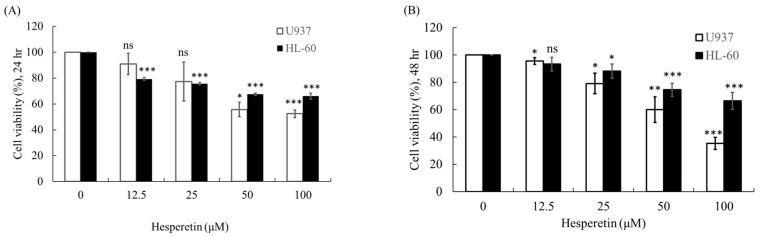
Effect of hesperetin on human leukemic cell viability. HL-60 and U937 cell lines (4 × 10^4^ cells/mL) were treated with hesperetin at 0, 12.5, 25, 50, and 100 µM for 24 h (**A**) and 48 h (**B**). Cell viability was evaluated using a CCK-8 assay. The *Y*-axis indicates the percentage of cell survival, and the *X*-axis indicates various concentrations of hesperetin. The mean ± SD of the three independent experiments performed in triplicate are shown. * *p* < 0.05; ** *p* < 0.01; *** *p* < 0.001 vs. the control group; ns, not significant; the results are representative of three independent experiments.

As presented in Figure 1A,B, U937 and HL-60 leukemic cell lines were evaluated for their response to hesperetin after incubation for 24 and 48 h, respectively. The results revealed that hesperetin dose-dependently reduced cell growth in both U937 and HL-60 cells (Figure 1A,B, *p* < 0.05). After 24 and 48 h of treatment, the half-maximal concentration (IC_50_) values were approximately 90.71 and 65.86 µM, respectively, in U937 cells and 260.7 and 198.7 µM, respectively, in HL-60 cells.

### 3.2. Analysis of Apoptosis and Cell Cycle Arrest

**Figure 2:**
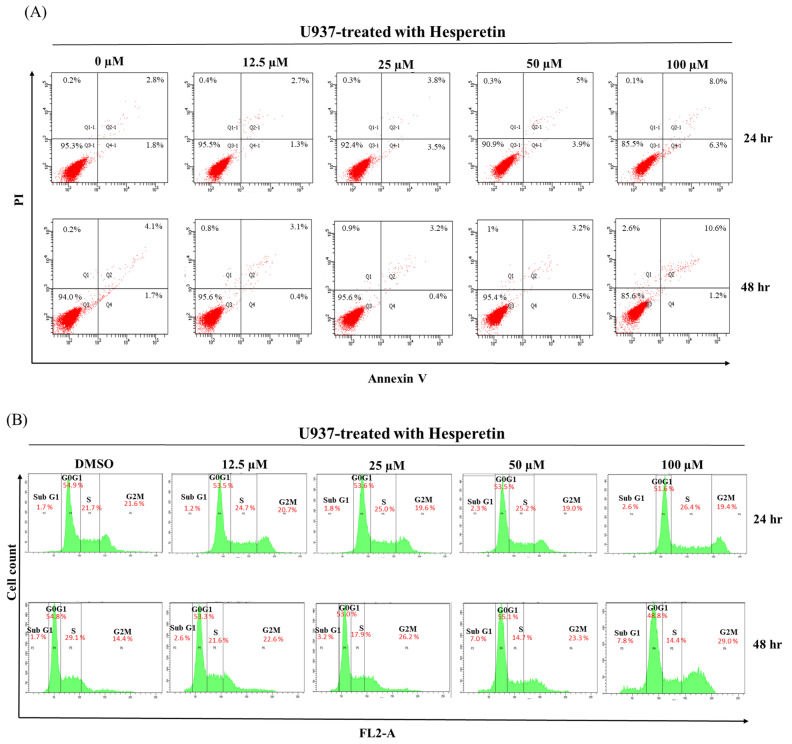

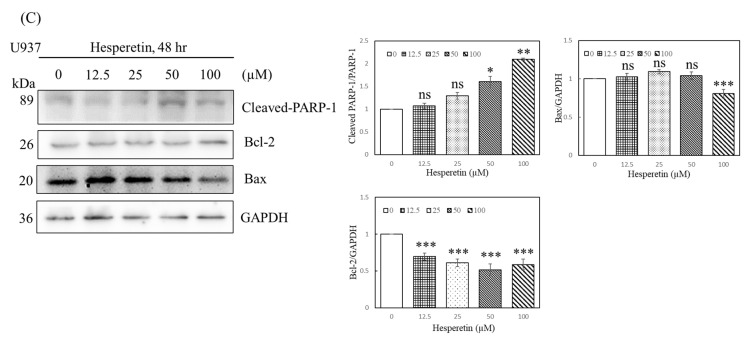
Effects of hesperetin compounds on apoptosis and cell cycle arrest of U937 cells. (**A**) U937 cell apoptosis was analyzed at 24 and 48 h by flow cytometry with annexin V-FITC/PI staining to distinguish early apoptotic (annexin V-FITC positive, PI negative; Q4-1 and Q4) from late apoptotic or necrotic cells (Annexin V-FITC positive, PI positive; Q2-1 and Q2). (**B**) The cell cycle was assessed using flow cytometry in U937 cells with or without hesperetin treatment. (**C**) U937 cells were treated with hesperetin at 0, 12.5, 25, 50, and 100 µM for 48 h. Cleaved-PARP-1, Bcl-2, Bax, and GAPDH expressions were analyzed with Western blotting by using the cell lysates. The mean ± SD of the three independent experiments performed in triplicate are shown. * *p* < 0.05; ** *p* < 0.01; *** *p* < 0.001 vs. the control group; ns, not significant; the results are representative of three independent experiments.

We determined whether hesperetin inhibits cell proliferation by inducing apoptosis. As presented in Figure 2A, the treatment of U937 cells with 12.5–100 µM hesperetin dose-dependently increased the number of apoptotic cells compared with the control group. Hesperetin induced a low degree of apoptosis, which led to leukemia cell death. Furthermore, Western blot analysis revealed that cleaved-PARP-protein levels increased in response to hesperetin treatment at various concentrations (Figure 2C, 50 and 100 μM, *p* < 0.05), and that the pro-apoptotic protein Bax protein slightly increased expression (Figure 2C, 100 µM, *p* <0.05). The expression of the anti-apoptotic protein Bcl-2 was also significantly decreased in a dose-dependent manner (Figure 2C, *p* < 0.05); its expression was significantly lower in hesperetin-treated leukemia cells than in control cells. Moreover, the Bax protein level was slightly lower in hesperetin (100 μM)-treated leukemia cells than in untreated control cells (Figure 2C, *p* < 0.05). We next determined the effect of hesperetin on the regulation of cell cycle distribution and apoptosis in U937 cells and analyzed their DNA content by using flow cytometry. As presented in Figure 2B, hesperetin treatment resulted in a low degree of S-phase arrest of U937 cells and simultaneously decreased the number of cells in the G1 phase in a concentration-dependent manner. Moreover, hesperetin significantly induced G2/M-phase arrest in U937 cells after 48 h incubation (29.0%).

### 3.3. Analysis of Autophagy

**Figure 3:**
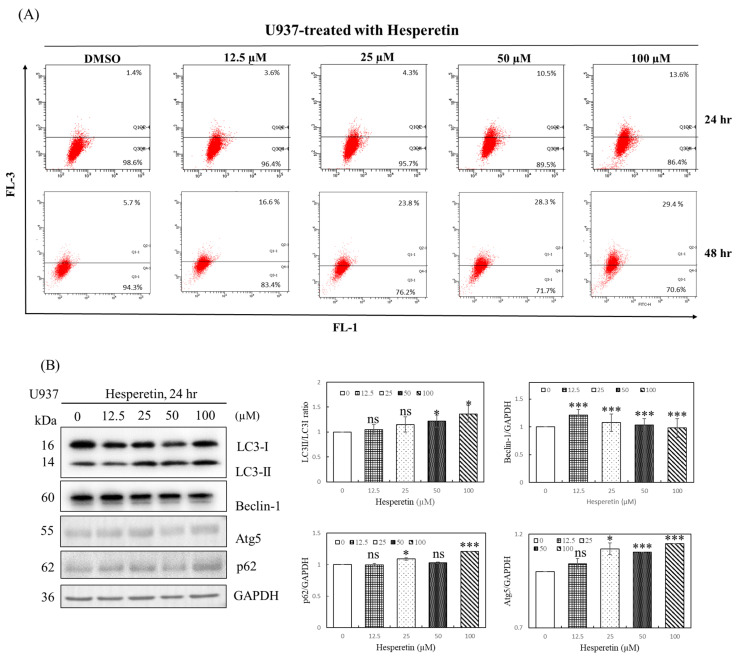

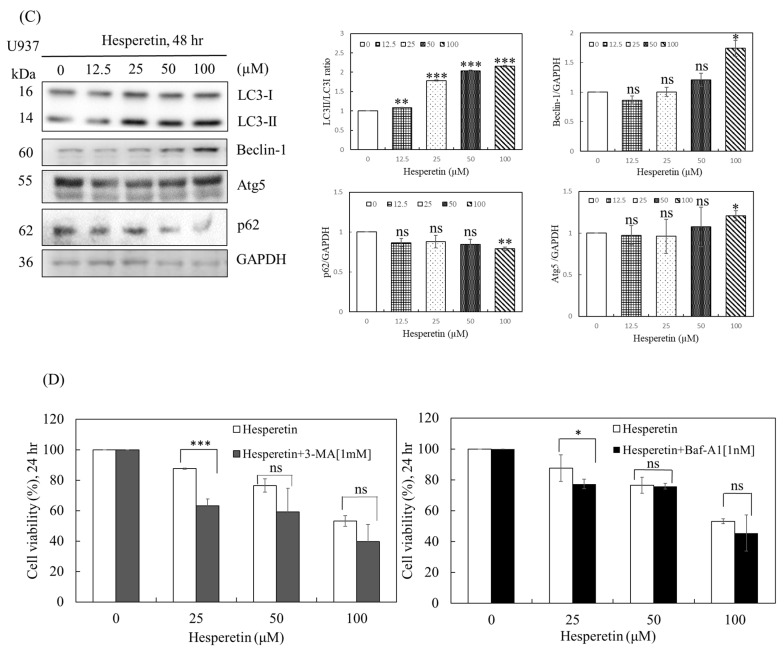
Effects of hesperetin on autophagy in U937 cells. (**A**) To evaluate the change in the number of AVOs in cells treated with hesperetin, the cells were treated with different concentrations of hesperetin at 24 and 48 h. Next, the cells were stained with acridine orange (1 μg/mL) at 37 °C for 15 min in the dark. The cells were analyzed using a FACScan flow cytometer. The data were analyzed using BD Cell Quest software. (**B**) LC3-I/II, Beclin-1, Atg5, p62, and GAPDH protein expression for 24 and 48 h (**C**) were analyzed with Western and semiquantified in representative results of the same pattern from three independent experiments are shown. (**D**) U937 cells were exposed to the indicated concentration of hesperetin in the presence or absence of autophagy inhibitor 3-MA (left panel) or Baf-A1 (right panel) for 24 h. Cell viability was assessed using the CCK-8 assay. The mean ± SD of the three independent experiments performed in triplicate are shown. * *p* < 0.05; ** *p* < 0.01; *** *p* < 0.001 vs. the control group; ns, not significant; the results are representative of three independent experiments.

As mentioned, a low degree of apoptosis was observed at 24 and 48 h of treatment with 12.5–100 µM hesperetin, indicating that other mechanisms modulate hesperetin-induced cell death. Autophagy may play a cell pro-death and pro-survival function in response to cellular stress [14,15]. Sequestosome 1 (SQSTM1; also known as p62) is a multifunctional protein that plays a critical role in autophagy. It is involved in the proteasomal degradation of ubiquitinated proteins. Cell survival can be significantly influenced by the modification of p62 levels in cells [16]. The conversion of LC3-I to LC3-II is also a vital marker of autophagy. LC3B-II is specifically localized to autophagic structures throughout the autophagic process, from the phagophore to the lysosome [13]. To identify the possible mechanism underlying hesperetin-induced cell death, we analyzed U937 cells treated with hesperetin for 24 and 48 h through acidic vesicular organelle (AVO) staining. The formation of AVO, stained by aggregated acridine orange in acidic compartments, was examined using flow cytometry to quantify autophagy in U937 cells. As presented in Figure 3A, hesperetin treatment for 24 h dose-dependently increased the percentage of acridine orange-accumulated cells in 3.6–13.6% of U937 cells. Moreover, hesperetin-induced autophagy significantly increased at 48 h (16.6–29.4%). Western blotting revealed that p62 protein levels decreased in response to hesperetin treatment at various concentrations, and that the protein expression of LC3B-II and the LC3II/LC3I ratio also significantly increased in a dose-dependent manner (Figure 3B, LC3II/LC3I ratio, 50 and 100 µM, *p* < 0.05; Figure 3C, 12.5 to 100 µM, *p* < 0.05, respectively). These findings suggested that the number of autophagosomes increased in response to hesperetin treatment, which are typically negatively regulated by p62 through its interaction with ubiquitin and the LC3 protein on autophagosomes. Furthermore, hesperetin dose-dependently increased the levels of the autophagy regulators Beclin-1 (Figure 3B, 12.5 to 100 µM, *p* < 0.05; Figure 3C, 100 µM, *p* < 0.05, respectively) and Atg5 (Figure 3B, 25 to 50 µM, *p* < 0.05; Figure 3C, 100 µM, *p* < 0.05, respectively). Therefore, our results suggest that the antiproliferation effect of hesperetin in U937 human leukemia cells may cause a transient autophagic response within 24 h and then induce apoptosis after 48 h. Moreover, we suggested that hesperetin can protect cancer cells during the transient period and may extend survival. Thus, hesperetin-induced autophagy may function as an adaptive response against apoptosis in the short term. Collectively, our findings suggest that autophagy may be a mechanism underlying hesperetin-induced cell death in U937 cells.

### 3.4. Effect of Autophagy Inhibition on Hesperetin-Induced Cell Death

Autophagy is a cell survival mechanism for cancer cells, depending on the cancer type, size, and microenvironment, and the inhibition of autophagy may result in increased apoptosis rates. Recent investigations have revealed that autophagy leads to cellular self-degradation, which is followed by apoptosis. As a result, pro-survival and pro-apoptotic roles are plausible. However, the effect of hesperetin-induced autophagy on cell death remains unclear. We evaluated the effect of autophagy inhibitors, 3-MA and Baf-A1, on hesperetin-induced cell death to determine whether hesperetin-induced U937 cell death can be attributed to autophagy. As presented in Figure 3D, 1 mM 3-MA or 1 nM Baf-A1 effectively inhibited hesperetin-induced autophagy, implying that hesperetin significantly induces autophagy in U937 cells. Thus, hesperetin can protect cancer cells during a transient period and may extend survival.

### 3.5. Hesperetin Modulates AMPK/Akt/mTOR Signaling Pathway

**Figure 4:**
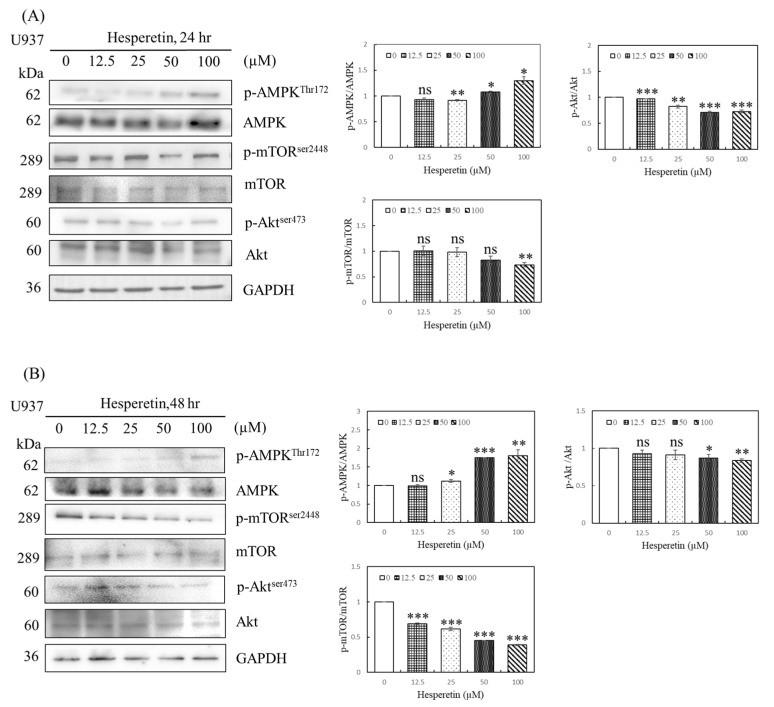
Effect of hesperetin on the AMPK/Akt/mTOR signaling pathway in U937 cells. U937 cells (5 × 10^5^) were treated with hesperetin at 0, 12.5, 25, 50, and 100 μM for 24 h (**A**) and 48 h (**B**). The expression of phospho-AMPK^Thr172^, AMPK, phospho-mTOR^Ser2448^, mTOR, Phospho-Akt^Ser473^, Akt and GAPDH was analyzed with Western blotting and semiquantified; representative results of the same pattern from three independent experiments are shown. The mean ± SD of the three independent experiments performed in triplicate are shown. * *p* < 0.05; ** *p* < 0.01; *** *p* < 0.001 vs. the control group; ns, not significant; the results are representative of three independent experiments.

The molecular mechanisms of apoptosis, cell cycle arrest, and autophagy during tumorigenesis are complex, and autophagy is crucial for cancer cells [9,11,12]. We evaluated the mechanism of action of hesperetin on human leukemia cells, focusing on autophagy, particularly the AMPK/Akt/mTOR pathway. AMPK/mTOR signaling was studied to examine whether hesperetin-induced AMPK activation contributes to apoptosis or autophagy. mTOR activation plays a vital role in autophagic regulation. In this study, U937 cells were treated with 0, 12.5, 25, 50, and 100 µM hesperetin for 24 and 48 h, and the expression of p-AMPK, p-Akt, and autophagy-related proteins was determined using Western blotting. We observed that compared with control cells, hesperetin significantly increased p-AMPK expression (Figure 4A,B, 25 to 100 µM, *p* < 0.05), reduced p-Akt expression (Figure 4A, 12.5 to 100 µM, *p* < 0.05; Figure 4B, 50 and 100 µM, *p* < 0.05, respectively), and decreased p-mTOR expression in a dose-dependent manner (Figure 4A, 100 µM, *p* < 0.05; Figure 4B, 12.5 to 100 µM, *p* < 0.05, respectively). These findings suggest that hesperetin induces autophagy by regulating the AMPK/Akt/mTOR pathway through AMPK activation and Akt downregulation.

## 4. Discussion

Hesperetin has anticancer activity for several cancers, such as carcinoid tumors, breast cancer, gastric cancer, and lung cancer [3,4,5,17,18,19,20]. In this study, hesperetin was found to inhibit leukemia cell viability in time- and dose-dependent manners. Moreover, hesperetin inhibited U937 cell growth by inducing a low degree of apoptosis, cell cycle arrest, and autophagy. Furthermore, treatment with 3-MA or Baf-A1 reversed hesperetin-induced cell death. These findings imply that hesperetin promotes early-stage apoptosis. Autophagy is a biological response to stress, and many anticancer drugs may stimulate autophagy as a pro-survival approach [21]. Accordingly, we suggest that hesperetin can protect cancer cells for a short period and may prolong their survival. Western blot analysis revealed that hesperetin decreased p-mTOR and p-Akt expression and increased p-AMPK expression. Overall, we demonstrated that hesperetin induced autophagy through the AMPK/Akt/mTOR pathway.

Apoptosis is one of the most common ways to prevent cancer development [22], and it is a key process with various internal and external regulators. Hesperetin has an inhibitory effect on different cancer cells. Chen et al. demonstrated that cells exposed to hesperetin exhibited a significant decline in viability through hesperetin-induced apoptosis [23]. Hesperetin treatment induced apoptosis in various types of cancer cells, including PC-3 prostate cancer cells, H522 lung cancer cells, U-251 cancer cells, and U-87 glioblastoma [24,25,26,27]. The Bax and Bcl-2, act as a promoter and an inhibitor of apoptosis, respectively. Both Bax and Bcl-2 as well as their ratio have been regarded as prognostic markers in various cancers [28,29]. By contrast, we observed that hesperetin induced a low degree of apoptosis of U937 cells by downregulating Bcl2 and upregulating cleaved-PARP and Bax, similar to the results of Shirzad et al. [30]. Furthermore, hesperetin induced G1 and G0/G1 arrest in different cancer cells, including MCF-7 breast cancer cells, HL-60 promyelocytic leukemia cells, and K562 chronic myeloid leukemia cells [31,32,33], as well as G2/M arrest in the SiHa cervical adenocarcinoma cell line and the U-251 glioblastoma cell line [8,27]. In the current study, hesperetin arrested cells in the G1 phase and resulted in a low degree of S-phase arrest of U937 cells. Furthermore, it significantly induced G2/M-phase arrest in U937 cells after 48 h treatment. Collectively, hesperetin induced leukemia cell death by inducing a low degree of apoptosis and cell cycle arrest. However, this result does not fully explain the inhibitory effect of hesperetin on leukemia cells, hinting at the presence of other underlying mechanisms.

Many studies have demonstrated that autophagy is a potential target of anticancer strategies. Autophagy is a conserved process that maintains cellular homeostasis by clearing damaged cellular components and balancing cellular survival and overall health [13]. However, few studies have explored the mechanisms underlying hesperetin-induced autophagy, particularly in cancer cells. For example, Saiprasad et al. found that hesperetin-induced autophagy in clone carcinogenesis [34]. Consistently, we demonstrated that hesperetin induced autophagy in U937 cells. Hesperetin dose-dependently increased the percentage of acridine orange-accumulated cells at 24 and 48 h. Classical, LC3, and Beclin-1 are critical and reliable autophagy markers. LC3 exists in two forms: cytosolic (LC3I) and membrane bound (LC3II), and increased LC3II levels are thought to be closely related to the extent of autophagosome formation [35]. Beclin-1 is required for the recruitment of other autophagic proteins during pre-autophagosomal membrane expansion and for the formation of the appropriate autophagosome structure [36]. In the current study, Western blot analysis revealed that hesperetin significantly decreased p62 levels and increased LC3-I to LC3-II conversion, Atg5 expression, and Beclin-1 expression in the U937 cell line, indicating its role as an autophagy inducer in U937 cells. Furthermore, hesperetin promoted early-stage apoptosis.

The interaction between apoptosis and autophagy is complex and serves various purposes. For example, mitophagy (mitochondrial autophagy) is mediated in embryonic cells to escape apoptosis by releasing cytochrome c from the mitochondria [37]. Our findings suggest that hesperetin-induced autophagy is an adaptive short-term response to maintain cellular energy homeostasis and avoid apoptosis, thereby serving a protective role. Apoptosis and autophagy are controlled by the interactions between Bcl-2 and Beclin-1. The Bcl-2 family of proteins have pro-apoptotic and anti-apoptotic functions. Bcl-2 activation is regulated by the posttranslational phosphorylation of Akt, mTOR, and p70S6K and inhibits apoptosis [38,39]. AMPK is a major metabolic modulator that restores the energy balance in response to physiological metabolic stress [15]. However, AMPK is also a growth factor, nutrient, and multiple kinase, and it can signal mTOR, which serves as a central control molecule. AMPK phosphorylation inhibits mTOR activity and activates autophagy [40,41]. mTOR is a mediator of the phosphatidylinositol-3-kinase (PI3K)-protein kinase B (Akt) signaling pathway, which is activated in response to metabolic and genotoxic stress and executes adaptive mechanisms for cell survival [42,43]. Akt, a serine/threonine kinase, regulates mTOR activity and plays a vital role in autophagy. Hesperetin activates AMPK in HepG2 cells, which can also activate autophagy through the AMPK/mTOR pathway to prolong the survival of specific dormant polyploidy giant cancer cells [44,45]. Wu et al. reported that hesperetin inhibits ECA-109 cells by suppressing the PI3K/Akt pathway and synergistically enhancing the antitumor effect [46]. Saiprasad et al. reported that hesperetin triggers autophagic markers mediated by PI3K/Akt in colon carcinogenesis, and Kim reported that hesperetin inhibits vascular formation by suppressing the PI3K/Akt pathway [47]. Yu et al. reported that AMPK is involved in VES-induced autophagy, and that crosstalk exists between AMPK and Akt/mTOR signaling [48]. By contrast, Li et al. reported that hesperidin treatment significantly decreased the myocardial infarct size, myocardial damage, and serum levels of creatine kinase-MB (CK-MB) and cardiac troponin I (cTnI) by downregulating p-mTOR, p-Akt, and p-PI3K in vitro [49]. He et al. also reported that hesperetin post-treatment prevented the cardiomyocytes effect by upregulating p-PI3K and p-Akt expression [50]. In our study, we observed that hesperetin significantly activated AMPK, inhibited p-Akt and p-mTOR, and subsequently modulated autophagy in leukemic U937 cells. Yu et al. also reported that VES initially triggers an AMPK-mediated autophagic process through AMPK/Akt/mTOR signaling [48]. Moreover, crosstalk exists between AMPK and the Akt/mTOR axis. Together, these findings suggest that AMPK modulates hesperetin-induced apoptosis and autophagy. These results indicate the potential mechanisms underlying hesperetin-induced autophagy and cell death in human leukemia cells by upregulating the AMPK/Akt/mTOR pathway. Therefore, we suggest that hesperetin is worthy of further studies to assess its potential as an adjuvant therapy, including in vivo experiments, which are warranted to establish hesperetin as a possible anti-leukemia treatment.

There are some limitations to our study. First, we only used one human leukemia cell line in this study because U937 cells are more sensitive to hesperetin treatment than HL-60 cells, according to our evidence that the HL-60 result is at much higher concentrations of hesperetin. Therefore, the results should be confirmed in other human leukemia cancer cell lines such as TF-1, K562, HL-60, KG-1, and THP-1. Second, to better represent and predict the therapeutic response in cancer, orthotopic xenograft mouse models will be used in further studies as valuable tools for improving our understanding in the study of anticancer drug responses. In addition, we need to establish hesperetin efficacy in patient-derived xenograft models before testing it in patients.

## 5. Conclusions

**Figure 5:**
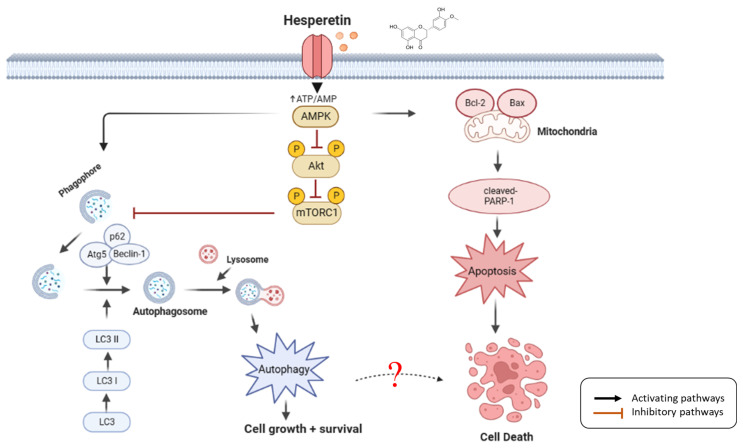
Schematic diagram of hesperetin molecular mechanism in leukemia cells. Hesperetin modulates AMPK/Akt/mTOR signaling and induces autophagy and delayed apoptosis by regulating the AMPK/Akt/mTOR pathway through AMPK activation, Akt and mTOR downregulation, inactivating and activating various target proteins, such as cleaved-PARP-1, Bcl-2, Bax, LC3-I/II, Beclin-1, Atg5, and p62; hesperetin promoted cell death in the human leukemic cell line U937 by inducing a low degree of slight apoptosis, cell cycle arrest, and autophagy, which increases the anticancer effect on leukemia (created with BioRender.com).

We demonstrated that hesperetin inhibited cell death in the human leukemic cell line U937 by inducing a low degree of apoptosis, cell cycle arrest, and autophagy. However, hesperetin may initially promote cell survival through autophagy and delaying apoptosis through the AMPK/Akt/mTOR pathway (Figure 5). Therefore, hesperetin-induced autophagy may function as an adaptive response against apoptosis in the short term. Hesperetin may thus be a potential adjuvant to antileukemia therapy.

## Data Availability

No datasets were generated for the preparation of this manuscript.
